# A hidden deadly venomous insect: First eco-epidemiological assessment and risk mapping of lonomism in Argentina

**DOI:** 10.1371/journal.pntd.0009542

**Published:** 2021-07-01

**Authors:** Milena Gisela Casafús, Marília Melo Favalesso, Micaela Andrea Gritti, Juan Manuel Coronel, Ana Tereza Bittencourt Guimarães, Maria Elisa Peichoto

**Affiliations:** 1 Consejo Nacional de Investigaciones Científicas y Técnicas (CONICET), Instituto Nacional de Medicina Tropical (INMeT)–ANLIS "Dr. Carlos G Malbrán", Puerto Iguazú, Misiones, Argentina; 2 Laboratório de Investigações Biológicas (LInBio), Universidade Estadual do Oeste do Paraná (UNIOESTE), Cascavel, Paraná, Brasil; 3 Cátedra de Biología de los Invertebrados, Universidad Nacional del Nordeste (UNNE), Corrientes, Corrientes, Argentina; 4 Programa de Pós-Graduação em Biociências, Instituto Latino-Americano de Ciências da Vida e da Natureza, Universidade Federal da Integração Latino-Americana, Foz do Iguaçu, Paraná, Brasil; Muséum National d’Histoire Naturelle, FRANCE

## Abstract

**Background:**

Envenomation by the South American *Lonomia* saturniid caterpillars, named lonomism, constitutes an emerging and somewhat neglected public health issue in Argentina and neighboring countries. Considering that there is an intricate relationship between environment and human health in such cases, this study aimed to analyze the eco-epidemiological profile of 40 accidents and 33 occurrences of *Lonomia* spp. in Misiones (Argentina) between January 2014 and May 2020.

**Methodology/Principal findings:**

We described the eco-epidemiological variables and characterized the abiotic scenario of such cases. Additionally, we obtained a density map that shows the punctual intensity of *Lonomia* records throughout Misiones. Most of the accidents occurred in the Department of Guaraní and involved male victims younger than 20 years old. The accidental/occasional occurrence of *Lonomia* spp. (considering both adult and caterpillar stages together) was significantly higher in the rural area, whereas only adult specimens were found in urban areas. We determined that the presence of this insect in Misiones is positively related to higher temperatures and solar radiation, and larger precipitation and evapotranspiration throughout the year.

**Conclusion/Significance:**

This study represents an initial step towards the global understanding of lonomism as a public health problem in Argentina. It provides a map of the risk level for this envenomation in Misiones, which could help authorities address public health policy efforts to implement sustainable strategies for prevention and response to this threat in Northeastern Argentina and neighboring regions.

## Author summary

Caterpillars of the genus *Lonomia* (Saturniidae: Hemileucinae) are considered the world’s most deadly lepidopterans. Due to the increasing number of cases and the lack of antivenom therapy, envenomation by these animals constitutes an emerging and somewhat neglected public health issue in Argentina and neighboring countries. Our research has determined the eco-epidemiological scenario of such an envenomation in the Argentine province of Misiones–mainly covered by the Atlantic Forest (a suitable biome for *L*. *obliqua*)–, and identified some predictors to occurrences/accidents with this insect. This study also provides the first map of the potential risk of lonomism in this province, which has several natural attractions visited by millions of tourists worldwide. We discussed our findings in light of immediate response from health authorities to outline preventive strategies for accidents and conservation of this venomous lepidopteran in its ecological niche.

## Introduction

Caterpillar envenomation is a global health threat that remains an underestimated problem, especially in countries with tropical climates [1]. Lonomism is the envenomation caused by the South American *Lonomia* caterpillars (Saturniidae: Hemileucinae), and it is characterized by a systemic hemorrhagic syndrome triggered by consumption coagulopathy. *Lonomia* envenomation case-fatality rate is six times higher than that observed for snake bites [2]. Because of its incidence and severity, lonomism is considered an important public health problem in tropical South America [3]. It is also of interest in travel medicine since some cases of such an envenomation have been reported in international travelers to this endemic area [4,5].

**Fig 1:**
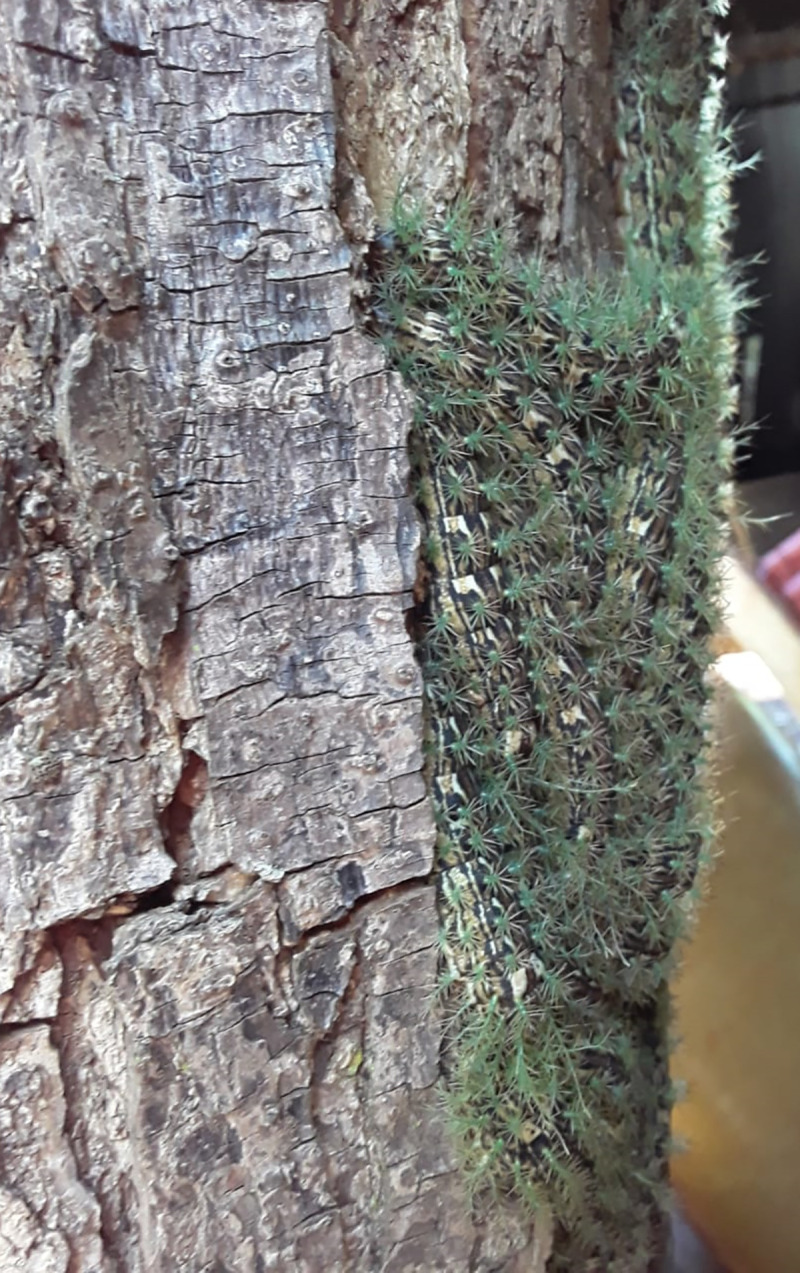
A colony of *Lonomia* sp. caterpillars on the trunk of a host tree.

Until the present moment, *Lonomia achelous* Cramer 1977 and *Lonomia obliqua* Walker 1885 are the species reported as responsible for envenomation in humans in South America [6,7]. Envenomation caused by *L*. *achelous* has been reported in Venezuela since 1967 [8], and this caterpillar is found in Venezuela, Bolivia, Colombia, Ecuador, French Guyana, Suriname, and North Brazil. *Lonomia obliqua* is mainly found in South Brazil and neighboring countries [6]. Envenomation by *Lonomia* occurs when a person accidentally comes into contact with a colony of caterpillars camouflaged on the tree trunks of their host species (Fig 1). Due to this contact, the animals are usually crushed, and the venom is injected subcutaneously–by the broken bristles–into the victim’s skin [9].

Since the 1980s, lonomism has been responsible for numerous deaths in Brazil, mainly in the southern region [7,9], and previously to the production of *Lonomia* antivenom by the Butantan Institute in São Paulo [10]. According to Azevedo [11], it is increasing in number annually and expanding to other areas in Brazil. Envenomation by *Lonomia* has also become a serious public health threat in Argentina over the last few years [12,13], especially in the Misiones province that exhibits several natural tourist attractions and shares the Atlantic Forest biome with Brazil. Favalesso et al. [14] demonstrated that this biome presents suitable areas for the presence of *L*. *obliqua*, and Misiones has the largest remnant of continuous Atlantic Forest. However, much of this area has undergone rapid land-use change during the last decades [15], decreasing the space between larvae and humans, which may be favoring the accidental contact.

It is worth to highlighting that lonomism can be considered a neglected public health problem requiring urgent actions in Argentina, since there is no availability of antivenom therapy in this country. Thereby, as the first step in this neglected and emerging health issue, it is necessary to lead an eco-epidemiological investigation to address public health policy efforts. Considering that lonomism is a type of envenomation whose etiological agent depends on specific environmental conditions, it is important to provide an ecological approach that helps health authorities outline preventive strategies for accidents and conservation of this lepidopteran in its ecological niche. Thus, in this study we aimed to analyze the eco-epidemiological profile of lonomism in Misiones (Argentina) between January 2014 and May 2020.

## Methods

### Ethics statement

We obtained epidemiological data from a collaborative effort with the SAMIC Hospital of Puerto Iguazú, according to an existing Cooperation Agreement between this institution and INMeT. Occurrence data were gathered from specimens collected *in situ* and semi-structured *ad-hoc* questionnaires authorized by the Ministry of Ecology and Renewable Natural Resources of Misiones (MERNR), according to authorization numbers 050-072/17, 016-036/18, 012/19, and 003/20. For all data, the information that identifies the patient/person was anonymized in the databases, and there is no need for ethical considerations according to national regulations.

### Study area

**Fig 2:**
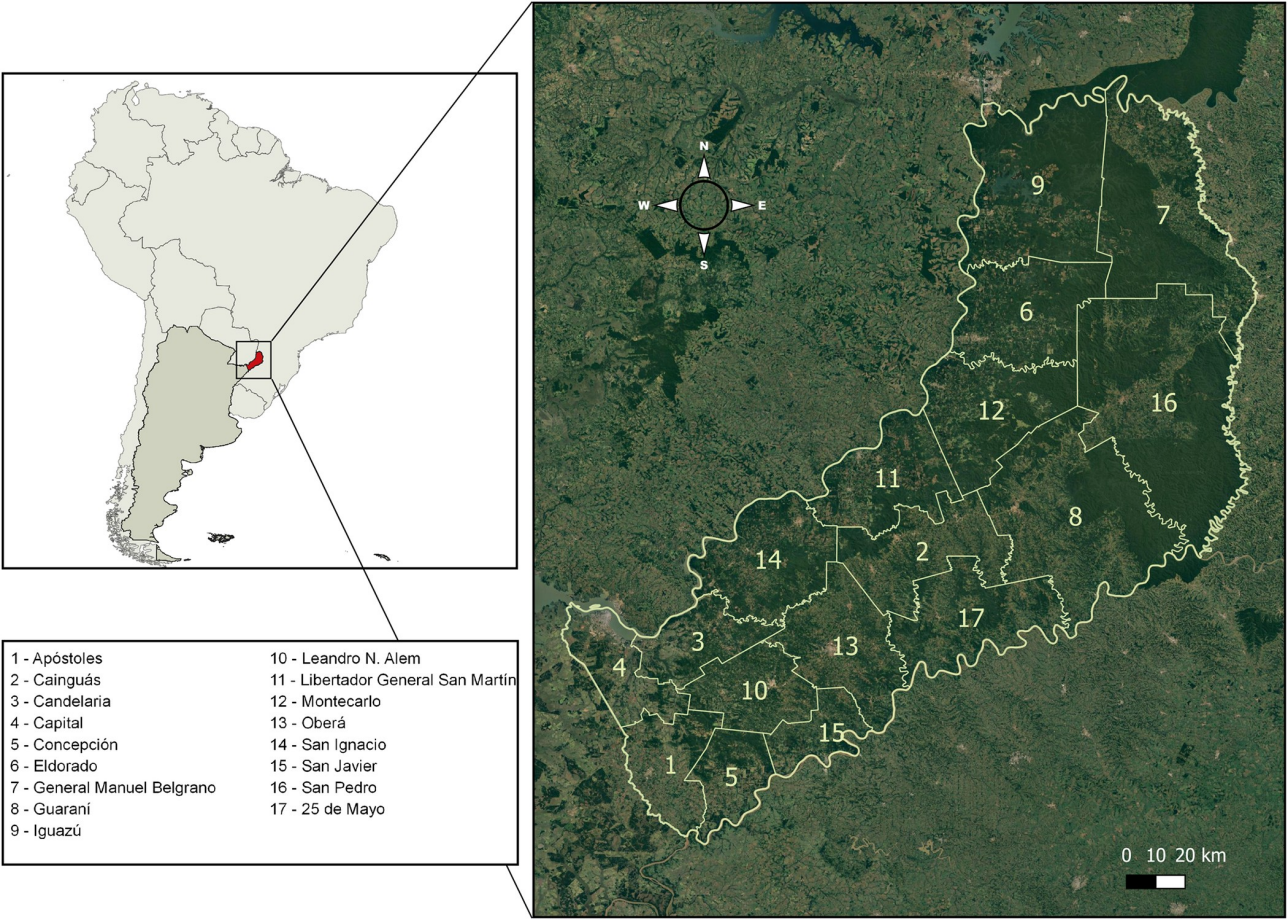
Study area, including the location of Misiones in South America and the distribution of the 17 departments within the province. The map was constructed in the QGIS 3.10.4-A Coruña, with layers downloaded from the National Institute of Geography (https://www.ign.gob.ar/NuestrasActividades/InformacionGeoespacial/CapasSIG).

We conducted this study in the province of Misiones (29,801 km^2^), northeastern Argentina (Fig 2). Misiones is politically organized in 17 departments, and the department is the smallest administrative unit for which there are reliable demographic data [16]. The climate is classified as Cfa in the Köppen system, corresponding to a subtropical humid climate without a dry season, with mean annual precipitation of 2,000 millimeters, uniformly distributed throughout the year, and mean annual temperature of 21°C [17].

Misiones possesses the largest remaining continuous area of Atlantic Forest (~10,000 km^2^) within the Upper Paraná Atlantic Forest ecoregion [18]. This region is one of Conservation Internationals Hotspots and is characterized by high biodiversity and endemism [19,20]. This forest type also occurs in southern Brazil and eastern Paraguay, although it is highly fragmented and occupies <6% of the original cover, mainly because of conversion to agriculture [21].

Although socioeconomic factors have driven increased deforestation in Misiones in the last decades [15,16], economic development in the province has lagged behind most of the other Argentinean provinces [22], leaving a relatively large portion of its area still covered by forests (~49%). Misiones has acquired a “green” profile as it is the Argentine province with the most significant percentage of protected territory (~17%), although these protected areas have different levels of effective management [17].

In addition to native forests, Misiones also includes urban and rural areas, creating a peri-urban area among them. According to Barsky [23], a peri-urban area is understood as a territorial complex of dynamic borders that includes elements of rural and urban land; it represents a transitional area in which boundaries are dynamic and depend upon the rhythm of urbanization. The peri-urban area represents an anthropogenic landscape in constant animal and vegetation domestication and change [24].

According to data from the last national census of population in 2010, Misiones had 1,101,593 inhabitants, with 73.76% living in urban localities, and 26.24% in rural areas [25]. Inhabitants in rural areas are engaged with horticulture, livestock farming, and industrial crops (yerba mate–*Ilex paraguayensis*–, tobacco and tea) [26]. Government subsidies also contribute to rapid increases in tree plantations, mainly including exotic species such as *Pinus* and *Eucalyptus*, and the native araucaria, *Araucaria angustifolia* [27]. However, there is also a variety of family agroforestry systems called “chacras” in Misiones, and they vary in design and management according to the use strategy of the environment by residents. Among the activities that characterize them, there are a diversity of crops, forestry production, citric production, extraction of timber and non-timber forest products; the products generated are for internal use of the domestic unit and/or sales in local markets [28].

### Lonomism cases

We used a total of 40 cases, reported from January 2014 to May 2020, spread across 15 localities in the Misiones province, Argentina. We obtained the data from the SAMIC public hospital in Puerto Iguazú city, the reference unit in the care of lonomism victims. Patient data were analyzed anonymously by the lead authors and a local assistant, after obtaining formal approval from the authorities of the SAMIC Hospital. For this study, we used specifically socio-demographic data, location, date, time, circumstances of the accident, and the anatomical area affected.

### Fieldwork and sampling

We conducted fieldwork in different localities of Misiones, from December 2017 to March 2020, based on information previously obtained from accident records, casual findings by residents, and analysis of the places with the highest probability of encountering *Lonomia* according to Favalesso et al. [29]. We applied a semi-structured *ad-hoc* questionnaire to local people, with in-depth interviewing and guided touring through the places of accident/occurrence of *Lonomia*. We collected moths at light and caterpillars from tree trunks when observed or notified by local people. Collected individuals were taken to the Entomology Laboratory of INMeT for further processing. Specimens were identified to genus level using entomological keys [30].

The following data were obtained *in situ*:

- the location coordinates (latitude/ longitude: decimal degree, datum WGS84),
- the popular name of the host plant (when possible, we collected some parts of the plant for taxonomic identification),
- and some environmental characteristics of the place that helped us to validate the type of environment.

We followed the descriptions previously reported for Misiones to classify the type of environment [17,23,24] and performed a visual interpretation of data using Google Earth. We used the number of houses and the type and percentage of land cover in a radius range of 100 meters around each point (we only consider the latter for the environments with ≤10 houses). The following is the environmental classification used in this study:

- urban: >20 houses,
- peri-urban: 10 < x ≤ 20 houses,
- rural: ≤10 houses with >50% of land area covered by agriculture/agroforestry,
- forest: without any house around the point but with ≥75% of land area covered by natural or seminatural forest.

We also recorded, through photos, the environmental characteristics and host plants where the accident/occurrence of *Lonomia* took place. Subsequently, we identified the host species with the collaboration of experts in plant taxonomy from the province of Misiones. The material used for identification was the physical sample and/or image.

### Eco-epidemiological analysis

We performed all statistical analyses using R software [31]. We described the qualitative variables by counts (n) and relative frequencies (%) of their categories, and we used the Goodness-of-Fit Chi-square test (α = 0.05), followed by post-hoc Pearson’s residuals, to compare these categories. Unknown data were excluded for all statistical analyses.

To contribute to characterizing the abiotic niche of *Lonomia* spp., we extracted climate data for each georeferenced point and for the month and year of sampling. These data were obtained from the TerraClimate dataset, using environmental layers with ~4 x 4km (1/24th degree) spatial resolution [32,33], and covering the period from 2014 to 2019. We extracted the following variables that were identified as a part of the *Lonomia* spp. niche in previous studies [14,34,35]: maximum temperature (°C), minimum temperature (°C), precipitation (mm), short wave radiation of descending surface (Wm^-2^), and evapotranspiration (mm/day). After that, we calculated the median and interquartile interval for all variables.

To show the punctual intensity of the occurrences of *Lonomia* spp. throughout the Misiones region, we obtained a record density map from the binned kernel density [36,37], a statistical method that uses the hotspots of point events to predict the incidence in the vicinity. We performed the analysis with two types of data: a) the exact coordinate of the individual’s sample; b) the centroid of the individual’s sampling city/municipality. We carried out the analysis through the R package “KernSmooth” [38].

## Results

From January 2014 to May 2020, we documented 40 accidents caused by larvae of *Lonomia* and 33 occurrences of this insect (10 in the larval stage plus 23 in the adult stage) in the province of Misiones, Argentina.

### Epidemiological profile

Socio-demographic aspects of lonomism victims and data regarding the location and circumstances of such accidents are shown in Table 1. Most of the accidents involved male victims younger than 20 years old. The accidents mainly occurred in the Department of Guaraní, during the daytime, while doing recreational or work activities in rural areas. The upper limb was the body area most significantly affected.

**Table 1:** Socio-demographic profile of victims of lonomic accidents (n = 40) in Misiones, Argentina.

Variable	Category	n	%	χ^2^	p-value
Sex	Female	11^b^	27.50%	8.10	0.0044
	Male	29^a^	72.50%	(df = 1)	
Age group	0–10	15^a^	37.50%	30.46	< 0.001
	11–20	11^a^	27.50%	(df = 6)	
	21–30	2^b^	5.00%		
	31–40	4^b^	10.00%		
	41–50	2^b^	5.00%		
	51–60	4^b^	10.00%		
	60–70	1^b^	2.50%		
	Unknown¤	1^b^	2.50%		
Area	Peri-urban	6	15.00%	5.17	0.0755
	Rural	17	42.50%	(df = 2)	
	Forest	13	32.50%		
	Unknown¤	4	10.00%		
Department	25 de Mayo	1^b^	2.50%	16.50	0.0113
	Alem	1^b^	2.50%	(df = 6)	
	Cainguás	8^b^	20.00%		
	Gral. Manuel Belgrano	6^b^	15.00%		
	Guaraní	10^a^	25.00%		
	Oberá	2^b^	5.00%		
	San Pedro	8^b^	20.00%		
	Unknown¤	4^b^	10.00%		
Circumstances	During work activity	12	30.00%	3.46	0.0630
	During recreational activity	23	57.50%	(df = 1)	
	Unknown¤	5	12.50%		
Time of the day	06:00–12:00h	12	30.00%	2.94	0.0863
	12:01–18:00h	22	55.00%	(df = 1)	
	Unknown¤	6	15.00%		
Affected body part	Lower limb	8^b^	20.00%	13.56	<0.001
	Upper limb	31^a^	77.50%	(df = 1)	
	Trunk	1	2.50%		

¤Category excluded for the analysis. The superscripted letters ‘a’ and ‘b’ refer to the highest and lowest classification of frequencies, respectively, according to the adjusted residual post-hoc test.

### Ecological aspects

The abiotic variables extracted from the georeferenced points of occurrence/accident cases of *Lonomia* spp. are shown in Table 2. The maximum and minimum temperature values were close to each other, indicating a moderate thermal amplitude throughout the area and period of study. The solar radiation level was high, and the variation range of this variable, as well as of precipitation and evapotranspiration, was wide.

**Table 2:** Median and interquartile interval of abiotic variables extracted from georeferenced points of occurrence/accident cases of *Lonomia* spp. in Misiones, Argentina.

Sample	Median	Interquartile interval
Maximum Temperature (°C)	28.4	[27.0–29.6]
Minimum Temperature (°C)	15.8	[14.7–17.3]
Precipitation (mm)	148.0	[117.0–210.8]
Evapotranspiration (mm/day)	115.0	[90.8–132.0]
Radiation (Wm^-2^)	206.0	[173.3–237.3]

**Fig 3:**
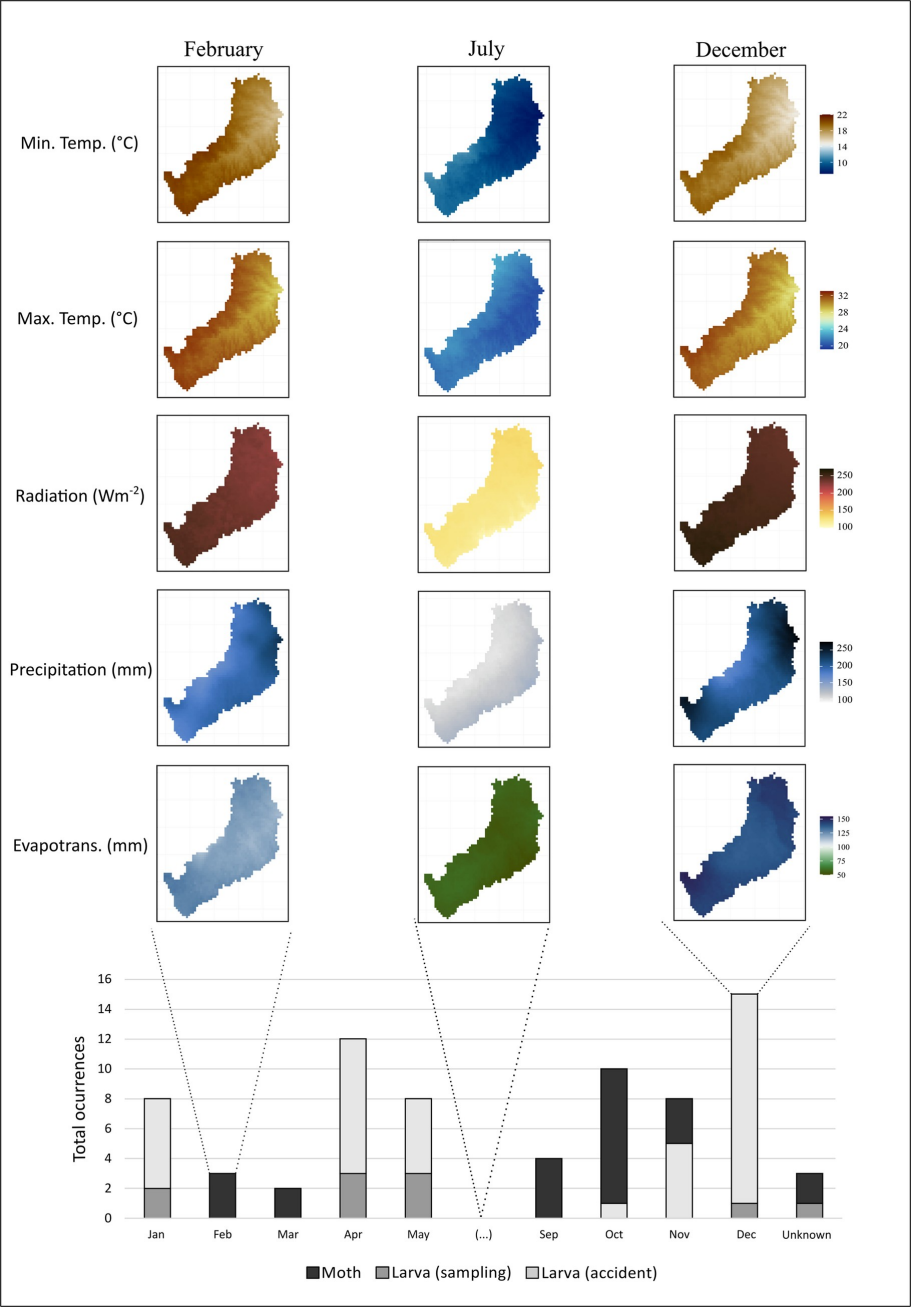
Accumulated occurrences of *Lonomia* spp. in the months of the study period (January 2014 to May 2020). Rasterized demonstration of evapotranspiration, rainfall precipitation, solar radiation, maximum and minimum temperatures in periods of high level of occurrences (January to May; September to December) and absence of occurrence (June to August). We produced the maps in R version 4.0.2 using climate rasters from TerraClimate (http://www.climatologylab.org/terraclimate.html) and layers downloaded from the National Institute of Geography (https://www.ign.gob.ar/NuestrasActividades/InformacionGeoespacial/CapasSIG) as basemap.

When we qualitatively evaluated the previous abiotic factors in relation to the occurrence/accident months, the frequency of *Lonomia* spp. larvae or adults was found to be positively related to the months with higher temperatures, characteristics of the summer and spring seasons, when solar radiation is also higher, as well as there are larger precipitation and evapotranspiration (Fig 3).

**Fig 4:**
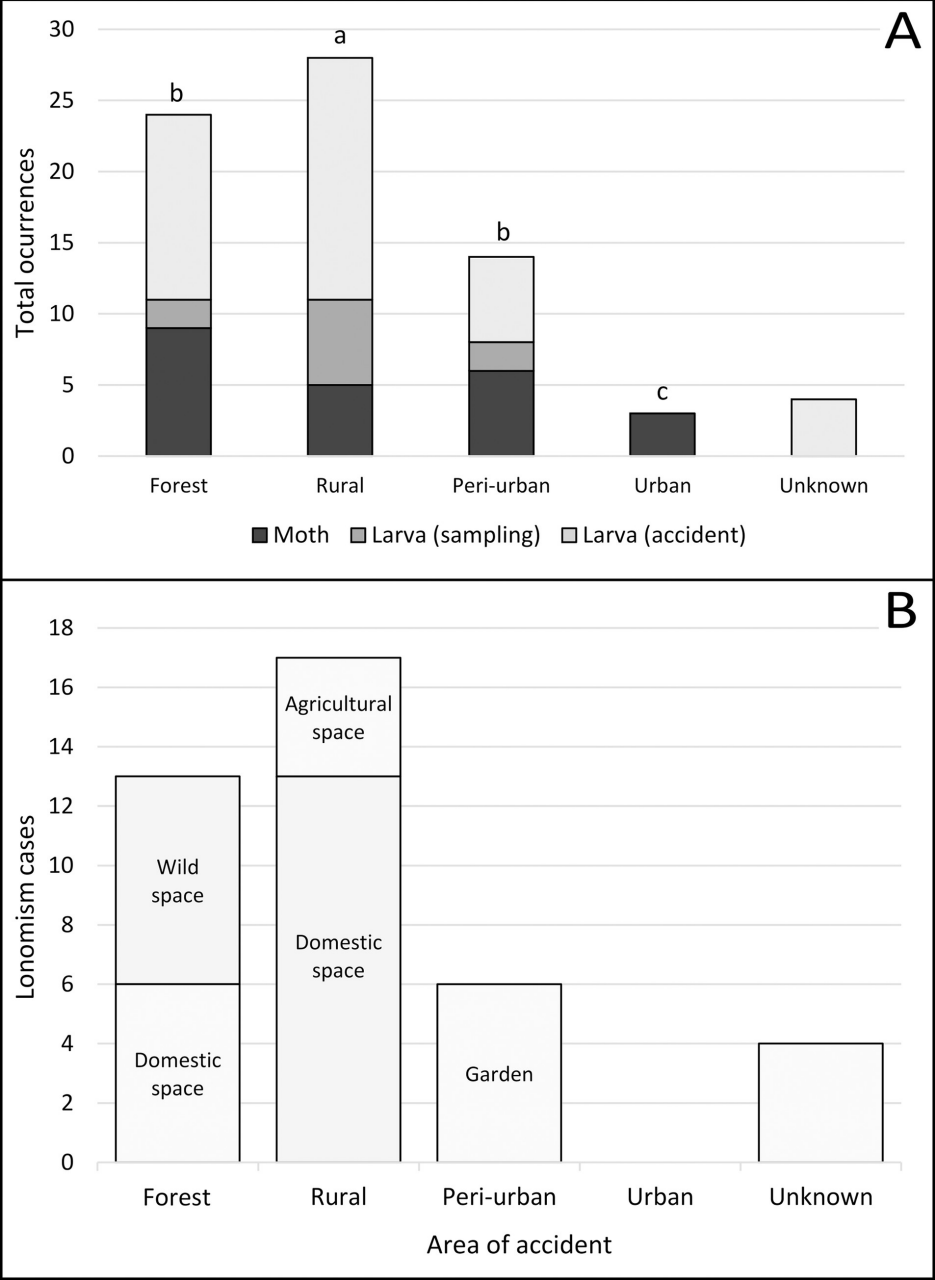
**A**- Distribution by area of accidental/occasional occurrence of *Lonomia* spp. in Misiones, Argentina. **B**- Distribution by area and specific space inside each area where lonomism cases happened in Misiones, Argentina. The superscripted letters ‘a’ and ‘b’ refer to the highest and lowest classification of frequencies, respectively, according to the adjusted residual post-hoc test.

**Fig 5:**
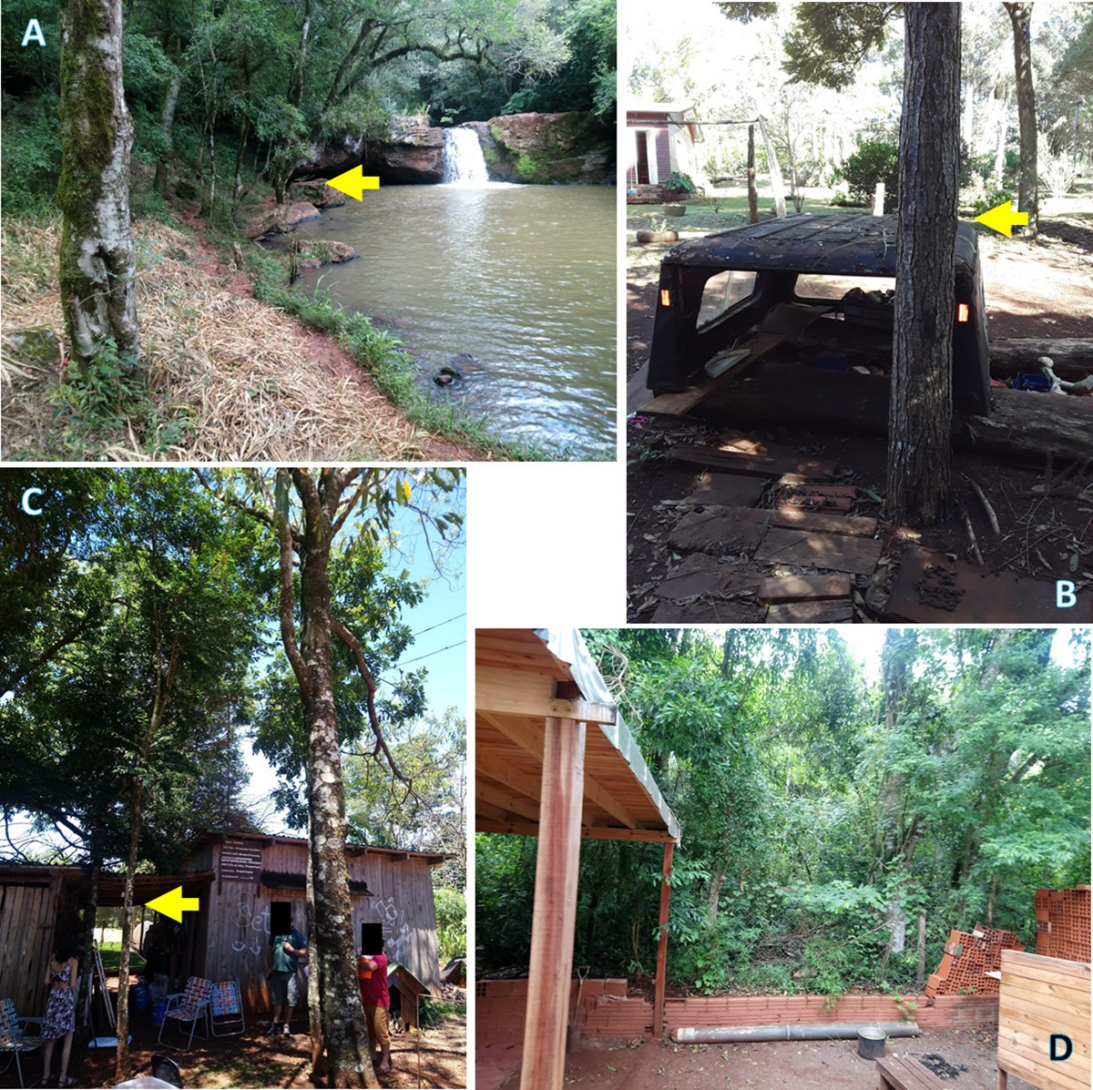
Environments where *Lonomia* spp. was found in Misiones, Argentina. **A**- A forest area. **B**- A domestic space inside a rural area. Note a metal scrap (commonly used by children to play) next to the host tree. **C**- A home garden in a peri-urban area. **D**- The backyard of the house (in a neighborhood of Puerto Iguazú city) where a moth of *Lonomia* was collected: note its proximity to the Iguazú National Reserve. In Fig 5A–5C, the yellow arrow indicates the tree hosting *Lonomia* caterpillars.

The accidental/occasional occurrence of *Lonomia* spp. was significantly higher in the rural area (χ^2^ = 21.73, df = 3, p <0.001), whereas only adult moths could be found in urban areas (Fig 4). In Fig 5, we show the typical environments where *Lonomia* specimens were found in this study. During the fieldwork, and whenever possible, the host plants for the larval stage of *Lonomia* spp. were registered/collected and later identified (Table 3).

**Table 3:** Host plants (n = 50) for *Lonomia* larvae in Misiones, Argentina.

Variable	Category	n	%	χ^2^	p-value
Species	*Albizia niopoides* (Spruce ex Benth.) Burkart*	2	4.0%	8.625	0.9983
	*Alchornea glandulosa* Poepp.*	1	2.0%	(df = 24)	
	*Annona emarginata* (Schltdl.) H. Rainer*	1	2.0%		
	*Annona* sp.*	2	4.0%		
	*Banara tomentosa* Clos*	1	2.0%		
	*Casearia decandra* Jacq.	1	2.0%		
	*Casearia sylvestris* Sw.*	1	2.0%		
	*Cedrela fissilis* Vell.*	1	2.0%		
	*Helietta apiculata* Benth.*	1	2.0%		
	*Lagerstroemia* sp.	2	4.0%		
	*Lonchocarpus campestris* Mart. ex Benth.*	1	2.0%		
	*Luehea divaricata* Mart.*	1	2.0%		
	*Machaerium* sp.*	1	2.0%		
	*Mangifera indica* L.*	1	2.0%		
	*Matayba elaeagnoides* Radlk.*	1	2.0%		
	*Nectandra lanceolata* Nees & Mart.*	1	2.0%		
	*Paulownia* sp.	1	2.0%		
	*Persea americana* Mill.*	4	8.0%		
	*Pouteria salicifolia* (Spreng.) Radlk.*	1	2.0%		
	*Prunus domestica* L.*	2	4.0%		
	*Pyrus communis* L.	1	2.0%		
	*Rubus ulmifolius* Schott*	1	2.0%		
	*Schefflera morototoni* (Aubl.) Maguire, Steyerm. & Frodin*	1	2.0%		
	*Sebastiania brasiliensis* Spreng.*	1	2.0%		
	*Styrax leprosus* Hook. & Arn.*	1	2.0%		
	Unknown¤	18	36.0%		
Family	Anacardiaceae*	1	2.0%	24.886	0.0515
	Annonaceae*	3	6.0%	(df = 15)	
	Araliaceae*	1	2.0%		
	Euphorbiaceae*	2	4.0%		
	Fabaceae*	4	8.0%		
	Lauraceae*	8	16.0%		
	Lythraceae	2	4.0%		
	Malvaceae*	1	2.0%		
	Melíaceae*	1	2.0%		
	Paulowniaceae	1	2.0%		
	Rosaceae*	4	8.0%		
	Rutaceae*	1	2.0%		
	Salicaceae*	3	6.0%		
	Sapindaceae*	1	2.0%		
	Sapotaceae*	1	2.0%		
	Styracaceae*	1	2.0%		
	Unknown¤	15	30.0%		
Status	Native*	17	34.00%	2.462	0.1167
	Exotic*	9	18.00%	(df = 1)	
	Unknown¤	24	48.00%		
Tree type	Fruit tree	13	44.00%	2.314	0.1282
	Non-fruit tree	22	26.00%	(df = 1)	
	Unknown	15	30.00%		

*At least one registered case of lonomism. ¤Category excluded for the analysis.

**Fig 6:**
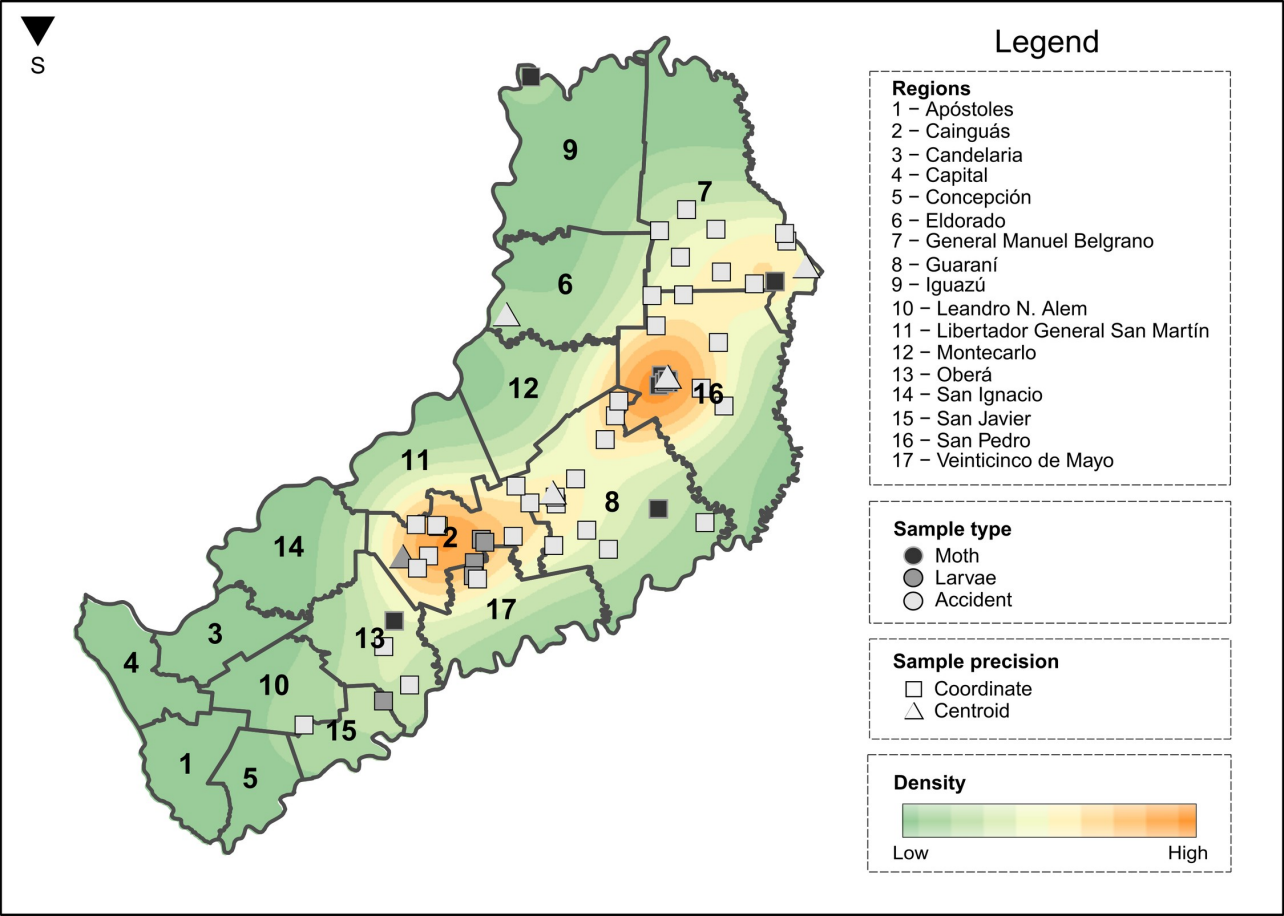
Lonomism risk map for Misiones, Argentina (January 26^th^, 2014—May 8^th^, 2020) using kernel density estimation. The map was constructed in R version 4.0.2, using layers downloaded from the National Institute of Geography (https://www.ign.gob.ar/NuestrasActividades/InformacionGeoespacial/CapasSIG) as basemap.

Finally, we found that the areas with the highest density of *Lonomia* spp. are in the central and northeast regions of Misiones (Fig 6), mainly in the departments of Cainguás, General Manuel Belgrano, Guarani, and San Pedro. The Iguazú point–where a moth was collected–is the one that has the most considerable distance from the others and corresponds to a place next to the Iguazu National Reserve (Fig 5D), a protected area of a representative environment of the Upper Paraná Atlantic forest and its biodiversity. It is important to emphasize that, even though we have used light-traps for sampling adults (once a month) and active searching for larvae in public spaces and/or the Apepu Station of the Argentine Iguazu National Park during a period of a year (November 2018 to November 2019), we could not yet record *Lonomia* in this protected area, which is an international tourist destination in Argentina that is visited by more than a million people per year.

## Discussion

In this study, we used an eco-epidemiological approach to characterize risk factors and environmental drivers for accidents with the venomous caterpillar *Lonomia* spp. in the province of Misiones (Argentina). Due to the increasing trend in the number of lonomism cases in recent years and the lack of antivenom therapy, these accidents have been a neglected problem. In general, the incidence of envenomation is the consequence of: a) the biology and behavior of the animal responsible for the envenomation, which explains its presence and abundance in a particular place and, b) human activities that can promote the victim’s contact with the animal, such as shown by Chippaux [39]. We reinforce that this study is the first to comprehensively explore this world’s most venomous Lepidoptera in Argentina, integrating Environmental Biology and Public Health through quantitative and qualitative analysis.

Following similar patterns that have taken place in cases of lonomism in Brazil [40], most accidents in Misiones occurred in young people and males, usually as a result of a rural activity (mainly recreation), and involved the upper limbs. They all happened during daylight conditions, which is related to the fact that *Lonomia* caterpillars stay together in groups on the trunk or lower branches of host trees during the day, moving toward the end of the branches to feed on the leaves at night [41]. On the trunk, they are almost imperceptible because their colonies exhibit mimicry characteristics in the host tree (Fig 1), which facilitates accidental contact with humans [40].

Guaraní is the department of Misiones that showed the highest number of accidents by *Lonomia*. This department is a region of the Atlantic Forest with a progressive fragmentation and separation of natural habitat patches [42], mainly due to land-use changes [43,44]. It is known that fragmentation of landscapes and habitat loss–driven by urbanization and/or climate change–can put wildlife species at risk of extinction and promote disease outbreaks in some scenarios [45]. Increasing landscape fragmentation promotes exotic plants [46] and arthropod invasion [47] in remnant forests, especially if the local disturbance is high. Land-use change has been considered a key factor influencing the incidence of envenomation by *Lonomia* spp. in southern Brazil [14], so our finding can be related to the highest deforestation rate that Guaraní has suffered in the last decades [42]. Coincidentally, this department, along with Cainguás, General Manuel Belgrano, 25 de Mayo, Oberá, and San Pedro, has experienced an increase in agricultural production, with a predominance of family farms [16,48].

The total number of *Lonomia* specimens recorded was significantly higher in rural areas, and we always found larvae outside the home, mainly in home gardens. Of the 40 accidents, nearly half (n = 17; 42.50%) occurred in rural areas. Surprisingly, although we have recorded larvae and adults of this lepidopteran in peri-urban areas, only adult moths could be found in urban areas. Although the sampling bias cannot be eliminated, it may indicate the beginning of the process of rural-to-urban migration, which occurred in southern Brazil more than a decade ago [34].

Of the several domestic and foreign tourist attractions in Misiones, the Iguazu Falls are the most visited natural attraction every year. Even though accident with *Lonomia* has not yet been registered in this area, travelers and local people should be cautious since we revealed the presence of this venomous lepidopteran in the city of Puerto Iguazú. Moreover, health professionals and authorities should be aware of the possibility of happening such an accident in this city, to adopt prevention and educational measures. It is worth mentioning that *Lonomia* specimens have already been sampled in Foz do Iguaçu. This Brazilian municipality borders Puerto Iguazú [49], and at least one accidental envenoming by *Lonomia* has been reported in the Iguazu National Park on the Brazilian side [5]. All these facts highlight the urgent need to prevent lonomism in the Iguazu Falls area and other Misiones areas.

The variables used here to describe the abiotic conditions for the presence of *Lonomia* spp. showed typical values for the environment of a neotropical formation of Atlantic Forest [50]. The climatic aspects, mainly air temperature and rainfall occurrence, have been considered determining factors for several biological aspects of *L*. *obliqua* [41]. Our results agree with the seasonal characteristics shown by Lorini [41] and support the fact that *Lonomia* spp. presents two generations per year (October and April) under the climatic conditions of Misiones. We also revealed that occurrences/accidents with *Lonomia* spp. are recorded in the months with higher temperature and greater precipitation, and the variation range of both variables are consistent with data reported by Gamborgi et al. [51] for the state of Santa Catarina, Brazil, bordering Misiones.

Considering that high humidity is essential for the development of *L*. *obliqua* [35,52], and for a better understanding of the water partitioning between surface and atmosphere, we evaluated the variability of precipitation and evapotranspiration in areas with the presence of *Lonomia* spp. Our results revealed a seasonal pattern for both variables, with the highest values concentrated in the hottest months of the year (when larvae and adults can be found), and the lowest values in the coldest months (when pupation probably occurs). Our findings confirm that evaporation depends on changes in solar radiation [53]; these two variables simultaneously showed the highest values in the study area.

The solar radiation variability reported here is consistent with that of Favalesso et al. [14], who described the potential niche of *L*. *obliqua* in Brazil. It is interesting to note that *Lonomia* spp. seems to prefer habitats with intense solar radiation. As mentioned in previous work [54], this abiotic variable can affect the performance of insect herbivores directly by increasing body temperature and thus the metabolic rate, or indirectly through alteration of either host plant quality or natural enemy activity. Although solar radiation is an important determinant of ambient air temperature, the dense vegetation of the Atlantic Forest biome absorbs–through its foliar pigments–most of the incident energy, having a great participation in maintaining the energy flow between the surface and the atmosphere [55]. This can explain the generally low-temperature variation in this biome [50].

Our study also found similarities between the maximum and minimum temperatures recorded in points of occurrence/accident cases of *Lonomia* spp. in Misiones and those reported by Favalesso et al. [14] for the predicted area for *L*. *obliqua* in Brazil. These temperature values are in the optimal range for the growth and development of most insect species [56]. It is also encouraging to compare the average monthly temperature obtained here with that found by Garcia [34] for the southern region of Brazil in months with the greatest notification of lonomic accidents; all these values are in the temperature range used for laboratory-rearing of *L*. *obliqua* [35,52].

Pupae are known to be the most vulnerable life stage of the lepidopteran life cycle due to an inability to escape from unfavorable abiotic conditions, predation, parasitoids, or microbes [57]. *Lonomia* larvae do not spin a pupal cocoon (which may provide some level of protection against moisture loss and subsequent desiccation and death), but rather form pre-pupae and naked pupae on the surface of the soil [41]. This soil is characteristically acidic in Misiones [58] and in the area predicted for *L*. *obliqua* in Brazil [14]. The low availability of phosphorus in Misiones soil [59] may help the elimination of inorganic phosphorus from pupal tissues, enhancing the splitting action of phosphorylases–which may be responsible for a phosphorolytical inactivation of coenzymes–in these tissues, until an equilibrium is reached at minimum metabolic rate [60].

As stated by Favalesso et al. [14], during the pupal diapause period of *L*. *obliqua* (i.e., during the coldest months of the year), the lower precipitation causes a decrease in the forest vegetation with biomass deposition on the soil, resulting in suitable temperature and humidity conditions for the pupal development under the soil surface layer. It is worth mentioning that Casafús et al. [12] showed that silt and clay particles were more common than sand particles in the soils of Misiones, where some lonomic accidents were registered. The small size and fine texture of the former result in small pores and a larger specific surface area for soil compaction and water adsorption, which may be necessary factors to the success of *Lonomia* pupal development, such as already demonstrated for other lepidopterans [61]. After that, when precipitation increases, the higher air temperature and humidity under a closed-canopy forest allow the species to reach the adult stage in which it reproduces and oviposits at the top of host trees. Later on, these hosts provide food to the larvae, which is the only feeding stage in the entire life cycle of this lepidopteran.

According to previous studies, the most common host trees for *L*. *achelous* and *L*. *obliqua* caterpillars are *Schefflera morototoni* [62] and *Platanus acerifolia* [52], respectively. In line with the fact that *Lonomia* caterpillars are quite polyphagous [63], we could verify their presence on *S*. *morototoni* and several other plant species, but not yet on *P*. *acerifolia*, even though this tree is common in some places of Misiones [64]. Although without statistical significance, we found here that native trees were more common hosts than exotic trees, and what calls our attention is that only fruit-tree species were included in the latter, which can be explained by the importance of such species for local people of Argentinian Atlantic Forest as a way of ensuring the provision of a variety of resources [24].

**Fig 7:**
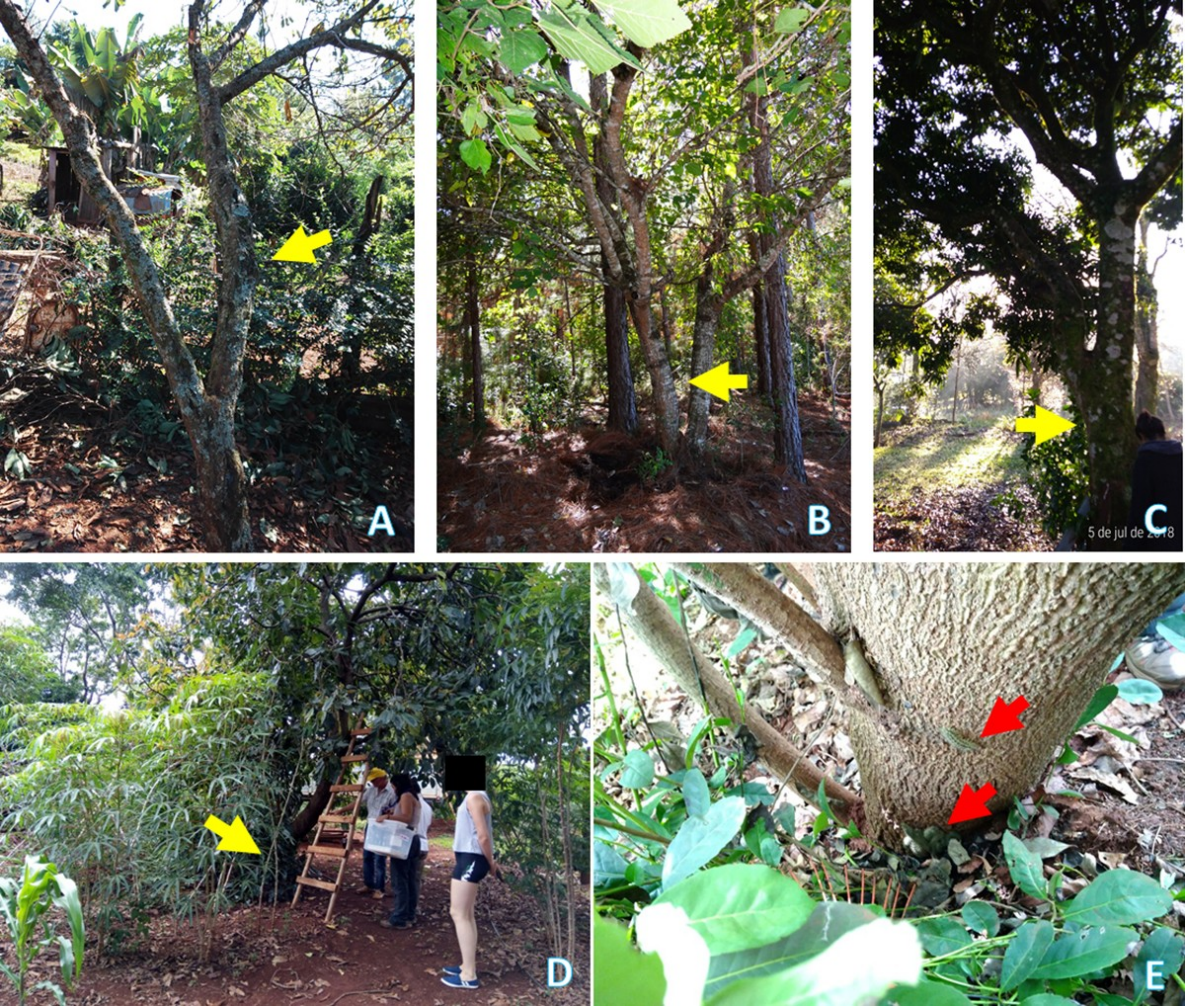
Fruit trees (indicated by yellow arrows) hosting *Lonomia* caterpillars involved in cases of lonomism in Misiones, Argentina. **A**- A plum tree with a fork that the victim used to climb the tree. **B**- A blackberry tree. **C**- A mango tree. **D**- A big palta tree hosting *Lonomia* in the lower part of its trunk. This part of the tree can be seen closer in **E**, where red arrows indicate the caterpillars.

According to Garcia [34] who reported *L*. *obliqua* especially in fruit trees in Southern Brazil, we registered several cases of lonomism in home gardens of rural and peri-urban areas of Misiones (Figs 4B and 7), where fruit species are prevalent [24]. However, contrary to expectations, this study did not show any significant difference between fruit and non-fruit hosts for *Lonomia* spp. This could be related to the fact that different plant species from the Atlantic Forest are grown in home gardens within Misiones for their local conservation [24]. Therefore, it can be assumed that these home gardens may represent an extension to the natural habitat of this lepidopteran.

It is essential to highlight that the sole previous study reporting plant hosts for *Lonomia* in Argentina is that of Pastrana [65], who recorded only *Erythrina crista-galli* L. (a native tree from the family Fabaceae) and *Pyrus communis* (an exotic tree from the family Rosaceae). Although its wide distribution in Argentina and even in Misiones (www.darwin.edu.ar), the former was not recorded here; however, we included four other plant species from the family Fabaceae in our list. Regarding *P*. *communis*, this is only one of several fruit host trees registered here, and *Persea americana* (from the family Lauraceae) prevailed over them, but without statistical significance. The palta tree was already previously described as hosting *L*. *obliqua* in Brazil [66].

Although evidence has shown that lactiferous plants suffer less from herbivory than other plant species [67], Santos et al. [68] identified *Lonomia* caterpillars feeding on rubber-trees (*Hevea brasiliensis* (Willd. ex A. Juss.) Muell. Arg.) in Northern Brazil. Herein, we found these lepidopterans on another lactiferous Euphorbiaceae species, *Sebastiania brasiliensis*, a native species widely distributed in Misiones and other states of Argentina (www.darwin.edu.ar). As the latex may work as a plant defense mechanism against insect herbivores [69], we suggest that *Lonomia* may have ways of sabotaging such a defensive role. The caterpillar *L*. *obliqua* contains protease inhibitors in its venomous structures [70], which may protect itself from toxic components in latex, such as cysteine and serine proteases [69,71].

Considering that the economy of Misiones is structured around forestry production and that plantation of *Eucalyptus* spp. is used for lumber and pulp production [48], we highlight that Bernardi et al. [72] have already shown that *L*. *obliqua* larvae are defoliators of the eucalypts, as well as other plantations, resulting in pest outbreaks [68]. Hence, monitoring the *Lonomia* population in Misiones is essential not only because it constitutes a public health hazard but also because it may cause economic losses in this province. It is also worth noting the ecological importance of this insect group, serving as prey of predatory organisms found in native ecosystems [49].

Overall, by showing the environmental factors associated with *Lonomia* spp. occurrence and the eco-epidemiological profile of human envenomation by this caterpillar, this study represents an initial step towards the global understanding of lonomism as a public health problem in Argentina. As such envenomation is restricted to Misiones, we present a risk map for this province. Such a map is key to the prevention of lonomism in local populations and also enable the prediction of risk for travelers who visit touristic areas in this endemic province of Argentina. It is important to point out that we aim to improve the accuracy of this risk map by taxonomic identification of the species involved in such cases, which is being investigated by the authors and will allow to trace further the ecological niche of this venomous insect.

We emphasize that the presence of *Lonomia* caterpillars in exotic host plants, the wide distribution of some native host trees, and the increased deforestation in Misiones constitute increased risks for the spread of this insect and, therefore, of lonomism. This reinforces that this envenomation constitutes an emerging public health issue of national concern that needs urgent attention and actions from the public health organs. Taking into account that the Atlantic Forest includes an adequate bioma for *Lonomia* [14,30], and aiming not to reach alarming epidemiological proportions in the future–such as already happened in Southern Brazil [73]–, it is also extremely important to adhere to the international efforts and national/provincial policy framework for conservation of the local Atlantic Forest hotspot biodiversity [20]. This may help guarantee that inhabitants and travelers in Misiones can take advantage of its natural surroundings without any health threat.

## Supporting information

S1 Script R code detailing the statistical analyses conducted in this study. (HTML)
