# Correction: A hidden deadly venomous insect: First eco-epidemiological assessment and risk mapping of lonomism in Argentina

**DOI:** 10.1371/journal.pntd.0011049

**Published:** 2023-01-18

**Authors:** Milena Gisela Casafús, Marília Melo Favalesso, Micaela Andrea Gritti, Juan Manuel Coronel, Ana Tereza Bittencourt Guimarães, Maria Elisa Peichoto

The tenth author’s initials are indexed incorrectly in PubMed. The correct initials are: Peichoto ME.
