# Supplementary material for: A hidden deadly venomous insect: First eco-epidemiological assessment and risk mapping of lonomism in Argentina
Source: PLoS Negl Trop Dis. 2021 Jul 1;15(7):e0009542. doi: 10.1371/journal.pntd.0009542 (PMC8279340; doi:10.1371/journal.pntd.0009542)
Supplement: S1 Script — (HTML) [file pntd.0009542.s001.html]

A hidden deadly venomous insect: first eco-epidemiological assessment and risk mapping of lonomism in Argentina


# A hidden deadly venomous insect: first eco-epidemiological assessment and risk mapping of lonomism in Argentina

#### MG Casafús, MM Favalesso, MA Gritti, JM Coronel, ATB Guimarães and ME Peichoto

## R packages

```
pacman::p_load(readxl, ggplot2, spaMM, lme4, MASS, sjPlot, pscl, corrplot, Hmisc, PerformanceAnalytics, mctest, fitdistrplus, GGally, ggExtra, sf, viridis, cartography, sp, ggspatial, binr, gganimate, plotly, gifski, magick, rgdal, rgeos, Rcpp, raster, ncdf4, RNetCDF, climateR, AOI, kableExtra, scico, tibble, cowplot, ggsn, reshape2, tidyverse, spatstat, maptools, KernSmooth, GISTool, dichromat, crayon)
```

## ***Table 1***: Socio-demographic profile of victims of lonomic accidents in Misiones, Argentina.

```
## --- data analysis --- ##
# Sex
s = c(11, 29)

sex = data.frame(Variable = c("Sex", ""),
                 Category = c("F", "M"),
                 n = s,
                 Residuals = c(chisq.test(s)$residuals %>% round(2)),
                 X2 = c(chisq.test(s)$statistic %>% round(2), paste0("(df = ", chisq.test(s)$parameter, ")")),
                 p =  c(chisq.test(s)$p.value %>% round(4), "")
                 )

# Age group
a = c(15, 11, 2, 4, 2, 4, 1)

age = data.frame(Variable = c("Age group", rep("", 6)), 
                 Category = c("0-10", "11-20", "21-30", "31-40", "41-50", "51-60", "61-70"),
                 n = a, 
                 Residuals = c(chisq.test(a)$residuals %>% round(2)), 
                 X2 = c(chisq.test(a)$statistic %>% round(2), paste0("(df = ", chisq.test(a)$parameter, ")"), rep("",5)), 
                 p = c(chisq.test(a)$p.value %>% round(4), rep("",6))
                 )

# Area
ar = c(6, 17, 13)

area = data.frame(Variable = c("Area", rep("", 2)), 
                 Category = c("Peri-Urban", "Rural", "Forest"),
                 n = ar, 
                 Residuals = c(chisq.test(ar)$residuals %>% round(2)), 
                 X2 = c(chisq.test(ar)$statistic %>% round(2), paste0("(df = ", chisq.test(ar)$parameter, ")"), ""), 
                 p = c(chisq.test(ar)$p.value %>% round(4), rep("",2))
)

# Departament
d = c(1, 1, 8, 6, 10, 2, 8)

departament = data.frame(Variable = c("Departament", rep("", 6)), 
                  Category = c("25 de Mayo", "Alem", "Cainguás", "Gral. Manuel Belgrano", "Guaraní", "Oberá", "San Pedro"),
                  n = d, 
                  Residuals = c(chisq.test(d)$residuals %>% round(2)), 
                  X2 = c(chisq.test(d)$statistic %>% round(2), paste0("(df = ", chisq.test(d)$parameter, ")"), rep("", 5)), 
                  p = c(chisq.test(d)$p.value %>% round(4), rep("", 6))
)

# Circumstances
c = c(12, 23)

circumstances = data.frame(Variable = c("Circumstances", ""), 
                  Category = c("During work activity", "During recreational activity"),
                  n = c, 
                  Residuals = c(chisq.test(c)$residuals %>% round(2)), 
                  X2 = c(chisq.test(c)$statistic %>% round(2), paste0("(df = ", chisq.test(c)$parameter, ")")), 
                  p = c(chisq.test(c)$p.value %>% round(4), "")
)

# Time of the day
t = c(12, 22)

time = data.frame(Variable = c("Time of the day", ""), 
                  Category = c("06:00 - 12:00h", "12:01 - 18:00h"),
                  n = t, 
                  Residuals = chisq.test(t)$residuals %>% round(2), 
                  X2 = c(chisq.test(t)$statistic %>% round(2), paste0("(df = ", chisq.test(t)$parameter, ")")), 
                  p = c(chisq.test(t)$p.value %>% round(4), "")
)

# Affected body part
af = c(8, 31)

affected = data.frame(Variable = c("Affected body part", ""), 
                  Category = c("Lower limb", "Upper limb"),
                  n = af, 
                  Residuals = chisq.test(af)$residuals %>% round(2), 
                  X2 = c(chisq.test(af)$statistic %>% round(2), paste0("(df = ", chisq.test(af)$parameter, ")")), 
                  p = c(chisq.test(af)$p.value %>% round(4), "")
)

## --- Table 1: Results --- ##
tab1 = rbind(sex, age, area, departament, circumstances, time, affected)

rownames(tab1) = NULL

tab1 %>% 
  kbl() %>% 
  kable_styling(bootstrap_options = c("striped", "hover", "condensed", "responsive"))
```

## ***Table 2*:** Median and interquartile interval of abiotic variables extracted from georeferenced points of occurrence/accident cases of *Lonomia* spp. in Misiones, Argentina.

```
## --- data analysis --- ##

## Shape of Misiones - Argentina 
## downloaded from:
## https://www.ign.gob.ar/NuestrasActividades/InformacionGeoespacial/CapasSIG

dep = st_read("departamento/departamento.shp")
prov = st_read("provincia/provincia.shp")
mis = prov[prov$nam == "Misiones",]

dep2 = st_crop(dep, mis)
misiones = dep2[dep2$fdc == "Ministerio de Ecología",]

## Occurence data
pontos = read_excel("amostragem.xlsx")
names(pontos) = c('id', 'life fase', 'x', 'y', 'month', 'year')

## --- TerraClimate data --- ## 
var = getTerraClim(AOI = misiones, param = c("tmax", "tmin", "prcp", "aet", "srad"),
                   startDate = "2014-01-01", endDate   = "2019-12-31")

## --- Extract the values for the points --- ##
l = list()

for(i in names(var)){
  for(j in c(1:dim(pontos)[1])){
    am = 
      if(pontos[j, 'month'] < 10){paste0("X", pontos[j, "year"], ".0", pontos[j, 'month'])
      }else{paste0("X", pontos[j, "year"], ".", pontos[j, 'month'])}
    
    l[[i]][[j]] = raster::extract(var[[i]][[am]], pontos[j, c(3,4)])
  }
}


## --- Table 2: Results --- ##
tab2 = data.frame(Sample = c("Maximum Temperature (ºC)", "Minimum Temperature (ºC)", "Precipitation (mm)", "Evapotranspiration (mm/day)", "Radiation (Wm^-2)"),
                  Median = c(round(quantile(l$tmax %>% unlist)[[3]],1),
                             round(quantile(l$tmin %>% unlist)[[3]],1),
                             round(quantile(l$prcp %>% unlist)[[3]],1),
                             round(quantile(l$aet %>% unlist)[[3]],1),
                             round(quantile(l$srad %>% unlist)[[3]],1)),
                  Q1 = c(round(quantile(l$tmax %>% unlist)[[2]],1),
                         round(quantile(l$tmin %>% unlist)[[2]],1),
                         round(quantile(l$prcp %>% unlist)[[2]],1),
                         round(quantile(l$aet %>% unlist)[[2]],1),
                         round(quantile(l$srad %>% unlist)[[2]],1)),
                  Q3 = c(round(quantile(l$tmax %>% unlist)[[4]],1),
                         round(quantile(l$tmin %>% unlist)[[4]],1),
                         round(quantile(l$prcp %>% unlist)[[4]],1),
                         round(quantile(l$aet %>% unlist)[[4]],1),
                         round(quantile(l$srad %>% unlist)[[4]],1))
)

tab2 %>% 
  kbl() %>% 
  kable_styling(bootstrap_options = c("striped", "hover", "condensed", "responsive"))
```

## ***Figure 3***: Accumulated occurrences of *Lonomia* spp. in the months of the study period (January 2014 to May 2020). Rasterized demonstration of evapotranspiration, rainfall precipitation, solar radiation, maximum and minimum temperatures in periods of high level of occurrences (January to May; September to December) and absence of occurrence (June to August).

```
## --- data analysis --- ##
n = list()
a = 0

for(i in c("02", "07", "12")){
  for(j in c(2014:2019)){
    n[[a + 1]] = paste0("X", j, ".", i)
    a = a + 1
  }
}

m = n %>% unlist()


## Min. Temp. (ºC) --
rtmin2 = var$tmin[[m[1:6]]] %>% 
  stack() %>% 
  raster::crop(misiones) %>% 
  raster::mask(misiones) %>% 
  stackApply(indices = rep(1, length(m)), fun = median, na.rm = T) %>%
  rasterToPoints() %>%
  tibble::as_tibble() %>% 
  ggplot2::ggplot() + geom_raster(aes(x = x, y = y, fill = index_1)) +
  scale_fill_scico(palette = "vik", direction = 1, limit = c(6, 22)) +
  coord_sf() + theme_bw() + labs(x = NULL, y = NULL, fill = "", title = "Min. Temp. (ºC) - February") 

rtmin7 = var$tmin[[m[7:12]]] %>% 
  stack() %>% 
  raster::crop(misiones) %>% 
  raster::mask(misiones) %>% 
  stackApply(indices = rep(1, length(m)), fun = median, na.rm = T) %>%
  rasterToPoints() %>%
  as_tibble() %>% 
  ggplot() + geom_raster(aes(x = x, y = y, fill = index_1)) +
  scale_fill_scico(palette = "vik", direction = 1, limit = c(6, 22)) +
  coord_sf() + theme_bw() + labs(x = NULL, y = NULL, fill = "", title = "Min. Temp. (ºC) - July")

rtmin12 = var$tmin[[m[13:18]]] %>% 
  stack() %>% 
  raster::crop(misiones) %>% 
  raster::mask(misiones) %>% 
  stackApply(indices = rep(1, length(m)), fun = median, na.rm = T) %>%
  rasterToPoints() %>%
  as_tibble() %>% 
  ggplot() + geom_raster(aes(x = x, y = y, fill = index_1)) +
  scale_fill_scico(palette = "vik", direction = 1, limit = c(6, 22)) +
  coord_sf() + theme_bw() + labs(x = NULL, y = NULL, fill = "", title = "Min. Temp. (ºC) - December")


## Max. Temp. (ºC) --

rtmax2 = var$tmax[[m[1:6]]] %>% 
  stack() %>% 
  raster::crop(misiones) %>% 
  raster::mask(misiones) %>% 
  stackApply(indices = rep(1, length(m)), fun = median, na.rm = T) %>%
  rasterToPoints() %>%
  as_tibble() %>% 
  ggplot() + geom_raster(aes(x = x, y = y, fill = index_1)) +
  scale_fill_scico(palette = scico_palette_names()[27], direction = 1, limit = c(18.5, 33.5)) +
  coord_sf() + theme_bw() + labs(x = NULL, y = NULL, fill = "", title = "Max. Temp. (ºC) - February") 

rtmax7 = var$tmax[[m[7:12]]] %>% 
  stack() %>% 
  raster::crop(misiones) %>% 
  raster::mask(misiones) %>% 
  stackApply(indices = rep(1, length(m)), fun = median, na.rm = T) %>%
  rasterToPoints() %>%
  as_tibble() %>% 
  ggplot() + geom_raster(aes(x = x, y = y, fill = index_1)) +
  scale_fill_scico(palette = scico_palette_names()[27], direction = 1, limit = c(18.5, 33.5)) +
  coord_sf() + theme_bw() + labs(x = NULL, y = NULL, fill = "", title = "Max. Temp. (ºC) - July")

rtmax12 = var$tmax[[m[13:18]]] %>% 
  stack() %>% 
  raster::crop(misiones) %>% 
  raster::mask(misiones) %>% 
  stackApply(indices = rep(1, length(m)), fun = median, na.rm = T) %>%
  rasterToPoints() %>%
  as_tibble() %>% 
  ggplot() + geom_raster(aes(x = x, y = y, fill = index_1)) +
  scale_fill_scico(palette = scico_palette_names()[27], direction = 1, limit = c(18.5, 33.5)) +
  coord_sf() + theme_bw() + labs(x = NULL, y = NULL, fill = "", title = "Max. Temp. (ºC) - December")

## Radiation (Wm^-2) --
rrad2 = var$srad[[m[1:6]]] %>% 
  stack() %>% 
  raster::crop(misiones) %>% 
  raster::mask(misiones) %>% 
  stackApply(indices = rep(1, length(m)), fun = median, na.rm = T) %>%
  rasterToPoints() %>%
  as_tibble() %>% 
  ggplot() + geom_raster(aes(x = x, y = y, fill = index_1)) +
  scale_fill_scico(palette = scico_palette_names()[16], direction = 1, limit = c(99, 265)) +
  coord_sf() + theme_bw() + 
  labs(x = NULL, y = NULL, fill = "", title = "Radiation (Wm^-2) - February") 

rrad7 = var$srad[[m[7:12]]] %>% 
  stack() %>% 
  raster::crop(misiones) %>% 
  raster::mask(misiones) %>% 
  stackApply(indices = rep(1, length(m)), fun = median, na.rm = T) %>%
  rasterToPoints() %>%
  as_tibble() %>% 
  ggplot() + geom_raster(aes(x = x, y = y, fill = index_1)) +
  scale_fill_scico(palette = scico_palette_names()[16], direction = 1, limit = c(99, 265)) +
  coord_sf() + theme_bw() + 
  labs(x = NULL, y = NULL, fill = "", title = "Radiation (Wm^-2) - July")

rrad12 = var$srad[[m[13:18]]] %>% 
  stack() %>% 
  raster::crop(misiones) %>% 
  raster::mask(misiones) %>% 
  stackApply(indices = rep(1, length(m)), fun = median, na.rm = T) %>%
  rasterToPoints() %>%
  as_tibble() %>% 
  ggplot() + geom_raster(aes(x = x, y = y, fill = index_1)) +
  scale_fill_scico(palette = scico_palette_names()[16], direction = 1, limit = c(99, 265)) +
  coord_sf() + theme_bw() + 
  labs(x = NULL, y = NULL, fill = "", title = "Radiation (Wm^-2) - December")


## Precipitation (mm) --

rprec2 = var$prcp[[m[1:6]]] %>% 
  stack() %>% 
  raster::crop(misiones) %>% 
  raster::mask(misiones) %>% 
  stackApply(indices = rep(1, length(m)), fun = median, na.rm = T) %>%
  rasterToPoints() %>%
  as_tibble() %>% 
  ggplot() + geom_raster(aes(x = x, y = y, fill = index_1)) +
  scale_fill_scico(palette = scico_palette_names()[21], direction = -1, limit = c(56, 240)) +
  coord_sf() + theme_bw() + 
  labs(x = NULL, y = NULL, fill = "", title = "Precipitation (mm) - February") 

rprec7 = var$prcp[[m[7:12]]] %>% 
  stack() %>% 
  raster::crop(misiones) %>% 
  raster::mask(misiones) %>% 
  stackApply(indices = rep(1, length(m)), fun = median, na.rm = T) %>%
  rasterToPoints() %>%
  as_tibble() %>% 
  ggplot() + geom_raster(aes(x = x, y = y, fill = index_1)) +
  scale_fill_scico(palette = scico_palette_names()[21], direction = -1, limit = c(56, 240)) +
  coord_sf() + theme_bw() + 
  labs(x = NULL, y = NULL, fill = "", title = "Precipitation (mm) - July")

rprec12 = var$prcp[[m[13:18]]] %>% 
  stack() %>% 
  raster::crop(misiones) %>% 
  raster::mask(misiones) %>% 
  stackApply(indices = rep(1, length(m)), fun = median, na.rm = T) %>%
  rasterToPoints() %>%
  as_tibble() %>% 
  ggplot() + geom_raster(aes(x = x, y = y, fill = index_1)) +
  scale_fill_scico(palette = scico_palette_names()[21], direction = -1, limit = c(56, 240)) +
  coord_sf() + theme_bw() + 
  labs(x = NULL, y = NULL, fill = "", title = "Precipitation (mm)- December")

## Evapotrans. (mm) --

rev2 = var$aet[[m[1:6]]] %>% 
  stack() %>% 
  raster::crop(misiones) %>% 
  raster::mask(misiones) %>% 
  stackApply(indices = rep(1, length(m)), fun = median, na.rm = T) %>%
  rasterToPoints() %>%
  as_tibble() %>% 
  ggplot() + geom_raster(aes(x = x, y = y, fill = index_1)) +
  scale_fill_scico(palette = scico_palette_names()[9], direction = -1, limit = c(44, 155)) +
  coord_sf() + theme_bw() + 
  labs(x = NULL, y = NULL, fill = "", title = "Evapotrans. (mm) - February") 

rev7 = var$aet[[m[7:12]]] %>% 
  stack() %>% 
  raster::crop(misiones) %>% 
  raster::mask(misiones) %>% 
  stackApply(indices = rep(1, length(m)), fun = median, na.rm = T) %>%
  rasterToPoints() %>%
  as_tibble() %>% 
  ggplot() + geom_raster(aes(x = x, y = y, fill = index_1)) +
  scale_fill_scico(palette = scico_palette_names()[9], direction = -1, limit = c(44, 155)) +
  coord_sf() + theme_bw() + 
  labs(x = NULL, y = NULL, fill = "", title = "Evapotrans. (mm) - July")

rev12 = var$aet[[m[13:18]]] %>% 
  stack() %>% 
  raster::crop(misiones) %>% 
  raster::mask(misiones) %>% 
  stackApply(indices = rep(1, length(m)), fun = median, na.rm = T) %>%
  rasterToPoints() %>%
  as_tibble() %>% 
  ggplot() + geom_raster(aes(x = x, y = y, fill = index_1)) +
  scale_fill_scico(palette = scico_palette_names()[9], direction = -1, limit = c(44, 155)) +
  coord_sf() + theme_bw() + 
  labs(x = NULL, y = NULL, fill = "", title = "Evapotrans. (mm) - December")

## --- Saving --- ##
ggsave("rtmin2.tiff", rtmin2, he = 15, wi = 20, un = "cm", dpi = 300)
ggsave("rtmin7.tiff", rtmin7, he = 15, wi = 20, un = "cm", dpi = 300)
ggsave("rtmin12.tiff", rtmin12, he = 15, wi = 20, un = "cm", dpi = 300)

ggsave("rtmax2.tiff", rtmax2, he = 15, wi = 20, un = "cm", dpi = 300)
ggsave("rtmax7.tiff", rtmax7, he = 15, wi = 20, un = "cm", dpi = 300)
ggsave("rtmax12.tiff", rtmax12, he = 15, wi = 20, un = "cm", dpi = 300)

ggsave("rrad2.tiff", rrad2, he = 15, wi = 20, un = "cm", dpi = 300)
ggsave("rrad7.tiff", rrad7, he = 15, wi = 20, un = "cm", dpi = 300)
ggsave("rrad12.tiff", rrad12, he = 15, wi = 20, un = "cm", dpi = 300)

ggsave("rprec2.tiff", rprec2, he = 15, wi = 20, un = "cm", dpi = 300)
ggsave("rprec7.tiff", rprec7, he = 15, wi = 20, un = "cm", dpi = 300)
ggsave("rprec12.tiff", rprec12, he = 15, wi = 20, un = "cm", dpi = 300)

ggsave("rev2.tiff", rev2, he = 15, wi = 20, un = "cm", dpi = 300)
ggsave("rev7.tiff", rev7, he = 15, wi = 20, un = "cm", dpi = 300)
ggsave("rev12.tiff", rev12, he = 15, wi = 20, un = "cm", dpi = 300)
```

The bar graph was designed using Microsoft Excel software.

The maps were related to the bar graph using the Inkscape software.

## ***Figure 4*:** **A**- Distribution by area of accidental/occasional occurrence of *Lonomia* spp. in Misiones, Argentina. **B**- Distribution by area and specific space inside each area where lonomism cases happened in Misiones, Argentina. The superscripted letters ‘a’ and ‘b’ refer to the highest and lowest (respectively) classification of frequencies according to the adjusted residual post-hoc test.

```
c(28, 24, 14, 3) %>% chisq.test()
chisq.test(c(28, 24, 14, 3))$residuals
```

Bar charts A and B were prepared using Microsoft Excel software.

Graphics A and B were merged into a single image using the Inkscape software.

## ***Table 3***: Host plants (n=50) for *Lonomia* larvae in Misiones, Argentina.

```
## --- data analysis --- ##
plant = read_excel("plant.xlsx") %>%
  as_tibble()

## Species --
spc = plant %>% filter(Variable == 'Species') %>% select(3)

spcd = data.frame(Variable = c("Species", rep("", 24)),
                 Category = plant %>% filter(Variable == 'Species')%>% select(2),
                 n = spc,
                 Residuals = c(chisq.test(spc)$residuals %>% round(2)),
                 X2 = c(chisq.test(spc)$statistic %>% round(3), paste0("(df = ", chisq.test(spc)$parameter, ")"), rep("", 23)),
                 p =  c(chisq.test(spc)$p.value %>% round(4), rep("", 24))
)

## Family --
fam = plant %>% filter(Variable == 'Family') %>% select(3)


fami = data.frame(Variable = c("Family", rep("", 15)),
                  Category = c(plant %>% filter(Variable == 'Family') %>% select(2)),
                  n = fam,
                  Residuals = c(chisq.test(fam)$residuals %>% round(2)),
                  X2 = c(chisq.test(fam)$statistic %>% round(3), paste0("(df = ", chisq.test(fam)$parameter, ")"), rep("", 14)),
                  p =  c(chisq.test(fam)$p.value %>% round(4), rep("", 15))
)

## Status --
st = plant %>% filter(Variable == 'Status') %>% select(3)

stat = data.frame(Variable = c("Status", ""),
                  Category = c(plant %>% filter(Variable == 'Status') %>% select(2)),
                  n = st,
                  Residuals = c(chisq.test(st)$residuals %>% round(2)),
                  X2 = c(chisq.test(st)$statistic %>% round(3), paste0("(df = ", chisq.test(st)$parameter, ")")),
                  p =  c(chisq.test(st)$p.value %>% round(4), "")
)

st = plant %>% filter(Variable == 'Status') %>% select(3)

stat = data.frame(Variable = c("Status", ""),
                  Category = c(plant %>% filter(Variable == 'Status') %>% select(2)),
                  n = st,
                  Residuals = c(chisq.test(st)$residuals %>% round(2)),
                  X2 = c(chisq.test(st)$statistic %>% round(3), paste0("(df = ", chisq.test(st)$parameter, ")")),
                  p =  c(chisq.test(st)$p.value %>% round(4), "")
)

## Tree type -- 
tree = plant %>% filter(Variable == "Tree type") %>% select(3)

tr = data.frame(Variable = c("Tree type", ""),
                Category = c(plant %>% filter(Variable == "Tree type") %>% select(2)),
                n = tree,
                Residuals = c(chisq.test(tree)$residuals %>% round(2)),
                X2 = c(chisq.test(tree)$statistic %>% round(3), paste0("(df = ", chisq.test(tree)$parameter, ")")),
                p =  c(chisq.test(tree)$p.value %>% round(4), "")
)

## --- Table 3: Results --- ## 
tab3 = rbind(spcd, fami, stat, tr)
rownames(tab3) = NULL

tab3 %>% 
  kbl() %>% 
  kable_styling(bootstrap_options = c("striped", "hover", "condensed", "responsive"))
```

## ***Figure 6***: Lonomism risk map for Misiones, Argentina (January 26th, 2014 - May 8th, 2020) using kernel density estimation.

```
## Data --
## Misiones
misiones2 = as_Spatial(mis)
citys_misiones = as_Spatial(misiones)

## Data points 
pnts = read_excel("pnts.xlsx")
coord = data.frame(pnts[,11], pnts[,10])

## Kernel denity estimate map --- 
## Density map
est = bkde2D(coord, 
             bandwidth = c(0.15, 0.15), 
             gridsize = c(1000L, 1000L)) 

## Transforming the density map into a raster
est.raster = raster(list(x=est$x1, y=est$x2, z=est$fhat))

## We are pairing the projection of the map with misiones
projection(est.raster) = projection(misiones2) 

## Cutting from the size of Misiones
result_raster = raster::mask(crop(est.raster, misiones2), misiones2)

## --- Result --- ##
## Statistical map for the manuscript ---
col_map = colorRampPalette(c("grey95", "grey65", "grey50"))(10) # 

# Plotting kernel
plot(c(-56.5, -52), c(-28.2, -25.5), axes = F, ann = F, type = "n") 
box(col = "black") 
plot(misiones, lwd = 2, border = NA, col = "grey95", add = T) 
plot(result_raster, add = TRUE, legend = F, col = col_map) 

# The divisions
plot(citys_misiones, lwd = 1.8, border = 'grey30', add = T) 

# Points
# Form and color for the points
pnts_form = ifelse(pnts$Coord == "Coordinate", 22, 24)
pnts_color = ifelse(pnts$ID == "Moth", "#FFFF00", 
                    ifelse(pnts$ID == "Larva", "#00FF00", "#FF3333"))

points(coord, pch = pnts_form, 
       bg = pnts_color, col = "black", cex = 1.3, lwd = 0.9)

# Division number names
centro = coordinates(citys_misiones)
citys_misiones$x = centro[,1]
citys_misiones$y = centro[,2]

names_city = data.frame(citys_misiones)
names_city$nam[12] = "Venticinco de Mayo"
names_city$cod = c(9, 6, 16, 12, 3, 7, 4, 2, 11, 8, 13,
                   17, 14, 10, 15, 5, 1)

text(names_city[,'x']-0.02, names_city[,'y']-0.09,  names_city$cod,
     cex=0.8, pos=3, col="black", bg ='white', font = 2) 

# Legends: 
text(-53.17, -25.45, "LEGEND", cex = 0.8, font = 2)

legend(-53.5, -25.58, title = expression(bold('      Regions')), cex = 0.6, 
       bg = NA,
       box.col = NA, title.adj = 0, adj = 0, text.font = 1,
       legend = c(paste0(sort(names_city$cod), " - ", str_sort(names_city$nam))))

legend(-53.5, -27.1, pch = 21, inset = .02, 
       legend = c("Moth", "Larvae", "Accident"), 
       pt.bg = c("#FFFF00",  "#00FF00", "#FF3333"), bty = "topleft", bg = NA, 
       cex = 0.6, pt.cex = 1.2, pt.lwd = 0.5,
       box.col = NA, title = expression(bold("Sample type")))

legend(-53.5, -27.53, legend = c("Coordinate", "Centroid"), pt.cex = 1,
       pt.lwd = 0.7,
       pch = c(22, 24), bg = NA, pt.bg = "grey60", cex = 0.6, 
       box.col = NA, title = expression(bold("Sample precision")))

plot(result_raster, legend.only = T, col = col_map,
     legend.args = list(text = expression(bold('Density ')), 
                        side = 3, line = 0.1, cex = 0.61), 
     axis.args = list(cex.axis = 0.61, col = "black", labels = c("Low", "High"), 
                      at = c(1.875618e-05, 1.461), lwd = 0.1), bg = "white",
     smallplot = c(0.655, 0.8, 0.235, 0.249), horizon = T,
     labels = F)
```

## Responsible for data analysis

**Ana Tereza Bittencourt Guimarães**


anatbguimaraes@gmail.com


/Ana-Guimaraes-27

**Marília Melo Favalesso**


mariliabioufpr@gmail.com


www.mmfava.com


Github: mmfava
